# Supplementary material for: Multidrug-Resistant Acinetobacter baumannii in Jordan
Source: Microorganisms. 2022 Apr 20;10(5):849. doi: 10.3390/microorganisms10050849 (PMC9144680; doi:10.3390/microorganisms10050849)
Supplement: Supplementary file 1 [file microorganisms-10-00849-s001.zip › microorganisms-1651089-supplementary.pdf]

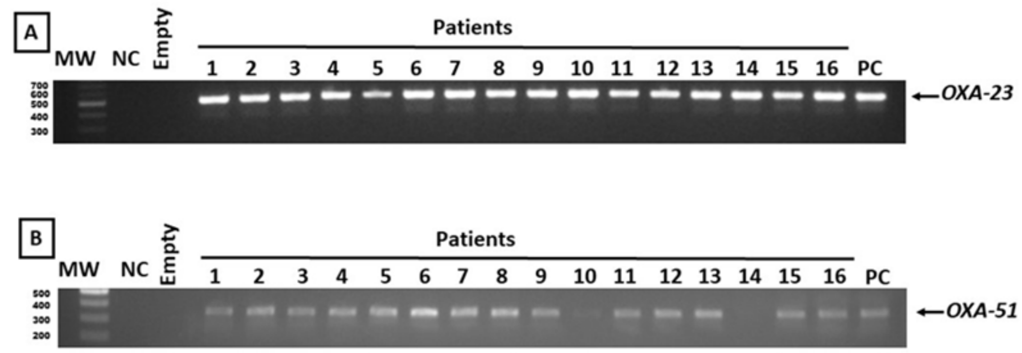

Figure S1. Analysis of PCR product by gel electrophoresis stained with ethidium bromide showing a band at around 501 base pairs for OXA-23 (A), and a band at around 353 base pairs for OXA-51gene (B). MW: Molecular weight ladder of 100 bp, NC: Negative control, Empty: empty control, and PC: Positive control.
